# The Emerging Role of NETs in Venous Thrombosis and Immunothrombosis

**DOI:** 10.3389/fimmu.2016.00236

**Published:** 2016-06-27

**Authors:** Andrew S. Kimball, Andrea T. Obi, Jose A. Diaz, Peter K. Henke

**Affiliations:** ^1^Section of Vascular Surgery, Conrad Jobst Vascular Research Laboratories, Department of Surgery, University of Michigan, Ann Arbor, MI, USA

**Keywords:** NETs, extracellular DNA, venous thromboembolism, venous thrombosis, sepsis, immunothrombosis

## Abstract

Venous thrombosis (VT), a leading cause of morbidity and mortality worldwide, has recently been linked to neutrophil activation and release of neutrophil extracellular traps (NETs) *via* a process called NETosis. The use of various *in vivo* thrombosis models and genetically modified mice has more precisely defined the exact role of NETosis in the pathogenesis of VT. Translational large animal VT models and human studies have confirmed the presence of NETs in pathologic VT. Activation of neutrophils, with subsequent NETosis, has also been linked to acute infection. This innate immune response, while effective for bacterial clearance from the host by formation of an intravascular bactericidal “net,” also triggers thrombosis. Intravascular thrombosis related to such innate immune mechanisms has been coined immunothrombosis. Dysregulated immunothrombosis has been proposed as a mechanism of pathologic micro- and macrovascular thrombosis in sepsis and autoimmune disease. In this focused review, we will address the dual role of NETs in the pathogenesis of VT and immunothrombosis.

## Introduction

Neutrophils (PMNs) have frequently been touted as the *pawns* of the immune system. As our knowledge of immune system grows and as our techniques for evaluating dynamic cell populations improve – we are learning that this could not be further from the truth. While a PMNs principle function remains as a key player in the front line of innate immunity and host defense against bacteria, they are proving to have a multifaceted role in coagulation and have also been implicated as major contributors in the pathophysiology of many systemic illnesses.

Until the early 2000s, the associations between PMN activation and systemic disease had not been well understood; but in March of 2004, Brinkmann et al. published a landmark study in *Science* (1), where they described a fragile fibrillar material extruded from PMNs in the presence of lipopolysaccharide (LPS) by transmission electron microscopy (TEM). In actuality, these fragile fibers were decondensed chromatin and DNA, as they stained strongly for DNA and histones, they were resistant to proteases, and they disappeared upon instillation of DNase. Bacteria were found to colocalize with the extruded DNA *in vivo* in a rabbit model of shigellosis and in human specimens of acute appendicitis. In summation, they demonstrated that these large webs of DNA trap bacteria and allow adjacent or connected PMNs to drive bactericidal activity with proteases and reactive oxygen species. Brinkmann et al. coined these nuclear extrusions “neutrophil extracellular traps” or NETs.

Since that time, there has been a flurry of exciting new work in the field of NET formation (NETosis). NETosis has been demonstrated to be a distinct form of cell death outside of necrosis and apoptosis (2). Also, more interestingly, NETs have been indicted in the pathophysiology of many systemic diseases, including venous thrombosis (VT) (3), sepsis (4, 5), trauma (6), cancer-related thrombosis (7), and autoimmune diseases (8–12). Despite the apparent widespread influence of NETs on disease, there remains a common theme throughout that NETs drive micro- or macrovascular thrombosis leading to ischemia and further injury (13, 14).

In this article, we will review the role of NETs in pathologic thrombosis. Specifically, we will review the findings of NET pathophysiology in murine models of VT, NETs in primate models and human studies of VT, and NETs in immunothrombosis.

## NETs in Murine VT Models

Murine models have been essential to our understanding of the role of NETosis in the pathophysiology of thrombosis. PMNs were first shown to be essential for immune-mediated microvascular thrombosis in a murine model of glomerulonephritis, in which CD11b^−/−^ or PMN-depleted mice were resistant to glomeruli thrombosis and renal failure (15). At that time, it was not widely recognized that NETs contributed to thrombosis; however, this changed in 2010, when Fuchs et al. showed that NETs caused platelet adhesion, activation, and aggregation (3). Stimulation of platelets with purified histones was sufficient for aggregation, and interestingly, DNase and heparin dismantled the NET scaffold and prevented thrombus formation. Brill et al. later demonstrated that NETs are principle effectors in an IVC stenosis model (16). In mice with uninterrupted IVC side-branches, levels of extracellular DNA increased in plasma 6 h after thrombus initiation. Citrullinated histone H3 (CitH3), an element of NETs’ structure, was present in thrombi and was frequently associated with the Gr-1 antigen. Furthermore, immunofluorescent staining of thrombi showed proximity of extracellular CitH3 and von Willebrand factor (vWF), a platelet adhesion molecule crucial for thrombus development in this particular model.

Neutrophils, monocytes, and NETs have also been found to affect the clotting cascade in murine models of thrombosis (17–20). For example, myeloid cells roll along the venous endothelium in a P-selectin-dependent manner and produce thrombogenic tissue factor (TF) in the IVC stenosis model (17). TF, then contributes to thrombin generation and extensive fibrin deposition along the vein wall. Despite this finding, TF alone was inadequate for thrombus propagation. Neutropenia, genetic ablation of Factor XII, and disintegration of NETs were all protective against thrombus propagation. Later, activated PMNs within the fibrin matrix were found to produce NETs that associate with secreted Factor XII, activate the intrinsic pathway, and lead to thrombus extension (17, 21). However, these conclusions were questioned, as during the same year, TF was also found to be secreted by PMNs in an inflammatory signaling and autophagy-dependent manner, with adherence to extruded NETs, activation of the extrinsic pathway, and eventual propagation of the thrombus (18, 22). Regardless, the presence of PMNs and/or monocytes at the endothelial interface has long been assumed to have a major role in VT and now appears unquestionable (17, 18, 23, 24).

Many proteins have been implicated in contributing to NETosis and thrombosis. In 2013, Martinod et al. demonstrated that the enzyme peptidyl arginine deiminase 4 (PAD4) – an enzyme essential for the citrullination and decondensation of chromatin was – not only essential for NET formation but also important for thrombus formation in the IVC stenosis model of murine VT (25). PAD4^−/−^ mice formed IVC thrombus in less than 10% of cases of IVC stenosis at 48 h compared to 90% in C57BL/6 controls (25). This antithrombotic tendency was rescued in the PAD4^−/−^ mice with adoptive transfer of wild-type PMNs. Conversely, in a stasis IVC ligation model of VT, either preemptive administration of DNase to wild-type mice or PAD4^−/−^ mice did not have an effect on thrombogenesis, suggesting a model-dependent effect (19).

Other proteins that have been implicated in NET formation and NET-associated thrombosis include cathepsin G, serine proteases, PMN elastase, P-selectin, high-mobility group box protein 1 (HMGB-1), platelet glycoprotein Ib, integrin beta-2, vWF, and platelet factor 4. Pharmacological inhibition or genetic deletion of cathepsin G increases mouse bleeding time, decreases platelet activation, and decreases circulating neutrophil–platelet conjugates (26). While the serine proteases and PMN elastase have been implicated in pathologic thrombosis and have been found to colocalize with NETs (27), it was recently demonstrated that PMN elastase is neither necessary for NET formation in mice nor essential for IVC thrombosis in murine IVC stenosis model (28). Indeed, it is likely that the prothrombotic effect of the serine proteases is better explained by their inhibition of tissue factor pathway inhibitor (TFPI) (29). Signaling through P-selectin by P-selectin glycoprotein ligand 1 (PSGL-1) is known to assist in PMN migration to sites of injury or inflammation (30), but beyond that, the P-selectin axis has now been postulated to promote NETosis in the setting of activated platelets (24). However, this conclusion was recently challenged in a study that demonstrated that HMGB-1 expressed by activated platelets was individually capable of activating PMNs *via* the receptor for advanced glycosylation end-products (RAGE) to induce NETosis (31). Similarly, in 2016, an *in vitro* study demonstrated activated platelets binding to vWF through glycoprotein Ib, in turn, linked to PMNs *via* integrin beta-2 and stimulated by platelet factor 4 led to NETosis independent of P-selectin (32). While our knowledge of NET formation continues to grow, the specific proteomic cascade that leads to NETosis remains contested. These findings also underline the heterogeneity and limitations of model systems as well.

Given that thrombi consist of significant quantities of DNA combined with proteins, thrombosis resolution and associated inflammation becomes less straight forward. We have explored this topic in our laboratory by examining toll-like receptor 9 (TLR9) function in VT resolution. TLR9 is a conserved pathogen-associated molecular pattern (PAMP) and damage-associated molecular pattern (DAMP) receptor that recognizes CpG DNA repeats and alerts the immune system to invading pathogens or local damage. In an IVC ligation model of VT (complete stasis), TLR9^−/−^ mice had significantly increased thrombus size at 2 and 8 days despite increased numbers of PMNs and monocyte/macrophages (33). Further, TLR9^−/−^ mice had increased apoptosis, citrullinated histones, PAD4, and neutrophil elastase; and reduced TFPI (19), suggesting that TLR9 is important for normal thrombogenesis and thrombosis resolution. Lastly, M1-like (CCR2+) monocyte/macrophages were decreased in TLR9^−/−^ thrombi, consistent with impaired inflammatory cell influx, and this divergence was corrected with adoptive transfer of TLR9^+/+^ bone marrow-derived monocytes with normalization of thrombus size (20). The new found composition of DNA-rich thrombi will likely have long-standing implications for future research in VT resolution.

## NETs in Primate and Human VT Studies

Although first thought to only occur in pathologic states, NETosis with release of extracellular DNA has been shown to occur in healthy individuals (34). Following exhaustive treadmill or cycling exercise, circulating levels of cell-free DNA (cfDNA) and myeloperoxidase (MPO) rise, and isolated PMNs from the circulation develop swollen nuclei and emanating DNA. A concomitant rise in circulating DNase occurs, suggesting that similar to the ongoing processes of fibrin formation and fibrinolysis, NETosis may be a tightly regulated and constantly ongoing homeostatic process. Dysregulation of NETosis and its relationship to thrombosis has been recognized in a variety of clinical scenarios: NETs are present in fresh thrombi from individuals with acute myocardial infarction (31, 35), they are found in high circulating levels in patients with severe trauma and microvascular thrombosis with acute lung injury (6); and in patients with thrombotic microangiopathies (TMAs) (8). Circulating cfDNA rises 24 h following chemotherapy in breast cancer patients, corresponding to peak in thrombin–antithrombin levels (36). This has been proposed as a potential causative mechanism for the high rate of thrombotic events experienced by individuals undergoing chemotherapy.

*Ex vivo* platelet activation studies with recombinant human histones demonstrate that not all histones are created equal in NETosis: only histone 3 (H3) and histone 4 (H4) induce functional platelet response (37). Recent advances in techniques of PMN isolation (38) and recognition of NETs in human pathologic thrombus specimens have improved the ability to study NETosis in humans (39).

The link between human venous thromboembolism (VTE) events and NETosis has been established in a handful of studies to date. Analysis of balloon occlusion-induced iliac thrombosis in a baboon model of VT demonstrated increased circulating NETs at 48 h post-thrombosis and persisting through 6 days (3). Consistently, extracellular DNA markers were present in the experimental thrombus. In 2013, a human study composed of healthy controls and symptomatic patients (swelling and leg pain) with and without VT examined circulating NET markers (40). Extracellular DNA and MPO were significantly elevated in symptomatic patients with VT compared to both groups of non-thrombosed patients. A direct correlation was also seen between common predictors of thrombosis including D-dimer, Wells score, and plasma DNA, suggesting that NETs may be useful in diagnostic evaluation. Development of more accurate methods to diagnose VT is of particular interest as the current “gold standard,” duplex ultrasound, is often limited in availability and in evaluation of central veins (41). In the same year, a similar case–control study of 195 individuals with and without VT examined nucleosomes and α-1 antitrypsin elastase (as a PMN activation marker) in relation to thrombosis. Levels of nucleosomes and PMN activation above the 80th percentile were associated with a threefold risk of VT (42).

The precise role of NETs in VT initiation, formation, and propagation, as well as optimal area to intervene in human VT remains relatively unknown. In a study evaluating unorganized, organizing, and organized thrombi from patients with either VT or PE, CD11b, and MPO positive cells were seen in organizing thrombi along with intra- and extracellular CitH3 and PAD4 (43). Unorganized and organized thrombi failed to demonstrate the similar histopathology, suggesting the predominant role of NETs during the inflammatory response and thrombus organization (43). Another study of 29 VT patients and controls demonstrated increased neutrophil adhesion and inflammatory cytokine profile among patients with residual vein obstruction and elevated D-dimer (44). *Ex vivo* studies of NETs related hypercoagulability in inflammatory bowel disease (IBD), and TMA patients have demonstrated efficacy of DNAse I in decreasing the thrombotic response, although this has yet to be shown in human VT (11, 12). To date, no clinical trial has targeted NETs in humans as a mechanism to prevent or treat VT.

## NETs in Immunothrombosis

While the ability to form thrombus in the face of vessel injury has long been known to be essential for the maintenance of hemostasis, until recently, it had not been recognized as an intravascular mechanism of immune defense. Engelmann and Massberg recently reviewed the mechanism of NET-mediated microvascular thrombosis and proposed it as a potential biological adjunct for containing uncontrolled infection, coining this process “Immunothrombosis” (45). In their review of the topic, they proposed four mechanisms by which immunothrombosis prevents the spread of infection (1) it captures and ensnares pathogens in the microvasculature and prevents dissemination, (2) it prevents distant tissue invasion by forming microthrombi in microvessels, (3) it concentrates pathogens in one area for bactericidal killing by innate immune cells, and (4) it recruits other immune cells to the site of inflammation for further bacterial killing. Indeed, coagulation has been previously postulated to play a role in host defense against bacteria (46, 47), but if left unchecked, immunothrombosis may contribute to significant systemic pathology.

Severe sepsis accounts for 10–40% of ICU admissions in the United States and carries a mortality rate of 20–50% (48, 49). NET-related immunothrombosis, cfDNA, and histones have been implicated in the morbidity and mortality of sepsis. In *in vitro* and *in vivo* models of sepsis, LPS has been shown to activate platelets and PMNs *via* toll-like receptor 4 (TLR4) to induce NETosis (31, 50, 51). Intravascular NETs can be digested by DNase to release cfDNA and histones, and further, cfDNA obtained from human specimens has been shown to be at ~150–300 bp sizes consistent with nucleosome units (52). In the same study, cfDNA levels in peripheral blood of patients with severe sepsis were found to be highly predictive of ICU mortality. In 2009, Xu et al. demonstrated that extracellular histones led to endothelial dysfunction, organ failure, and death in animal models of sepsis (4). Specifically, histone administration to mice resulted in neutrophil activation, endothelial toxicity, acute lung injury, and microvascular thrombosis. Interestingly, antihistone antibodies and activated protein C (APC) improved mortality rates in sepsis models in these mice. In addition, NETosis has also been linked to prothrombotic activity because NET-associated enzymes break down TFPI (29), while also enhancing antifibrinolytic activity because cfDNA inhibits plasmin-mediated fibrin degradation (13, 14, 28). Further, H3 levels were found to correlate with ICU mortality and were inversely correlated with antithrombin and platelet levels (53). Structurally, NET-induced immunothrombosis leads to more sturdy thrombi with less permeability and decreased susceptibility to lysis, although this may be overcome with DNase (54). In a murine cecal ligation and puncture model of sepsis (CLP), DNase given at 6 h after injury reduced organ damage and mortality (55). Lastly, bacterial resistance to NET trapping has been demonstrated *in vitro* and has been postulated to contribute to widespread immunothrombosis in the septic host leading to disseminated intravascular coagulation (DIC) (56, 57).

In addition to its inflammatory role in sepsis, NETs and immunothrombosis have also been implicated in autoimmune disease. Phosphatidylserine (PS) and TF-bearing microparticles (MP) were found to be markedly increased in the sera of IBD patients (11). Increased TF-bearing MPs correlated with increased NETosis, markedly shortened coagulation times, and increased levels of fibrin, thrombin, and factor Xa. Similarly, in an *in vitro* study of antineutrophil cytoplasmic antibody-associated vasculitis (ANCA-AV or AAV), C5a-primed PMNs treated with ANCA IgG-released NETs and TF-bearing MPs contributing to a prothrombotic state, and interestingly, this was remedied partially by DNase treatment (58). TMAs, more commonly known as thrombotic thrombocytopenic purpura (TTP), hemolytic uremic syndrome (HUS), and DIC, are characterized by microvascular thrombosis and coagulopathy. *In vitro* studies of TMAs demonstrate that the sera of TMA patients are incapable of degrading NETs due to a deficiency of DNase I and this may contribute to widespread microvascular thrombosis and organ dysfunction (12). Intriguingly, supplementation of TMA sera with recombinant human DNase I reestablished NET degradation capability. Lastly, PMNs from patients with antiphospholipid antibody syndrome (APS) were inclined for spontaneous NETosis and thrombin generation, and further, APS sera and APS-isolated IgG stimulated NETosis in control PMNs (59). These findings highlight the prothrombotic role of NETs in autoimmune-related immunothrombosis.

Finally, NETs have also been implicated in multiple organ failure related to severe trauma. In 2013, Abrams et al. examined a cohort of 52 patients with severe non-thoracic blunt trauma and correlated circulating histone levels with acute lung injury and sequential organ failure assessment scores (SOFA) (6). Circulating histone levels increased dramatically immediately following trauma and high levels correlated with acute lung injury, SOFA scores, endothelial injury, and coagulation activation. In the same study, with translational animal models of blunt trauma, circulating histone levels corresponded with organ edema, hemorrhage, microvascular thrombosis, and neutrophil congestion. Multiple organ dysfunction syndrome (MODS) is a leading cause of delayed mortality following major trauma and has been correlated with circulating cfDNA levels (52). Inherently, future research should look to address NETosis and immunothrombosis as potential therapeutic targets for preventing immune-mediated MODS after severe injury.

## Summary, Integration, and Potential Translation

**Figure 1:**
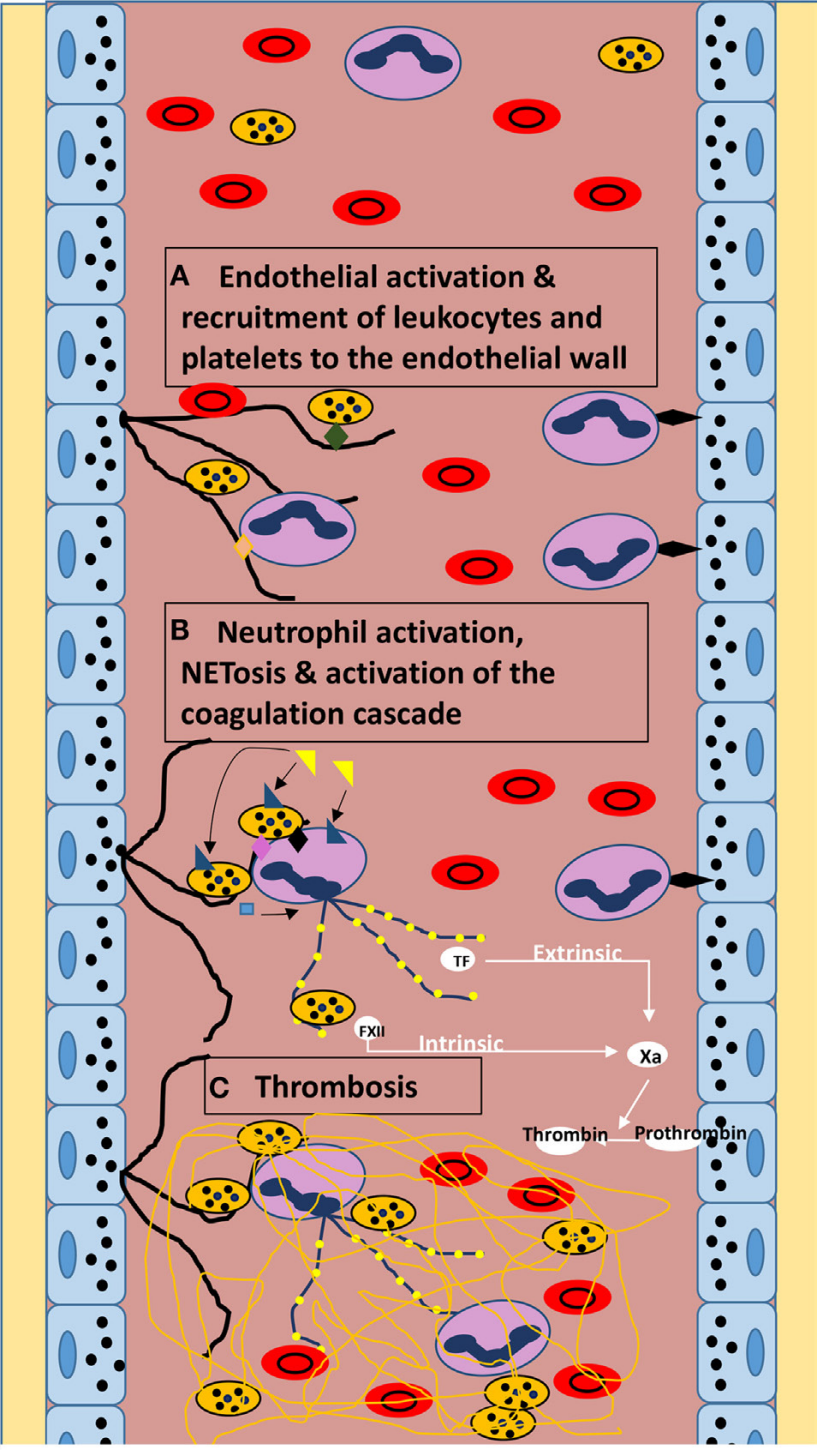
**Diagram of theoretical mechanism of NET-mediated microvascular thrombosis in sepsis**. **(A)** Sepsis-induced endothelial activation leading to the release of vWF (black lines) from endothelial cell Weibel–Palade bodies provoking the recruitment of leukocytes and platelets to the endothelial wall *via* vWF A1 domain–beta-2 integrin interaction (vWF–PMN interaction; orange diamond) and vWF A1 domain–glycoprotein Ib interaction (vWF–platelet interaction; green diamond). PMNs are also recruited to the endothelium *via* P-selectin-to-PSGL-1 interaction (black diamond) inducing neutrophil rolling. **(B)** Neutrophil and platelet activation by LPS (yellow triangle) *via* TLR4 (blue triangle). Platelet activation leading to the release of PF4 (blue square) and expression of P-selectin (black diamond) and HMGB-1 (pink diamond) further stimulate NETosis [purple lines of decondensed chromatin with attached histones (yellow circles)]. NETosis also leads to autophagy-induced release of TF, and NETs further stimulate FXII activation, inciting both the intrinsic and extrinsic coagulation cascades. **(C)** Thrombin activation by factor Xa leads to the conversion of fibrinogen to fibrin (yellow lines) culminating in microvascular thrombosis.

Over the past 12 years, since its discovery, NETosis has been catapulted to the forefront of innate immunity research. Nowhere is its effect more relevant in human disease than in its implication in immune-mediated micro- and macrovascular thrombosis (Figure 1). In this review and others, NETs have been shown to have a significant role in pathogenic thrombosis through platelet and PMN recruitment to the endothelial wall, subsequent activation and NETosis, and proteomic activation of the intrinsic and extrinsic coagulation cascades, ultimately leading to thrombosis (3, 13, 14, 16, 17, 31, 32, 45, 50, 60, 61).

This unique development in the etiology of immune-mediated thrombosis affords novel targets for the prevention of pathologic thrombosis in a susceptible patient (Table 1) (4, 25, 55, 62–70). One obvious target for the prevention of NET-mediated VTE is PAD4 inhibition (25, 60). Enzymatic inhibition of PAD4 would prevent NETosis, and hopefully, its prothrombotic effects as well; however, this may also leave the host susceptible to bacterial infection (71). DAMP-mediated NETosis by HMGB-1 is also a potential therapeutic target. HMGB-1 is released from damaged cells and expressed on the surface of activated platelets and leads to immune system activation *via* RAGE, TLR2, and TLR4 (5, 72, 73). HMGB-1-mediated PMN activation subsequently contributes to microvascular thrombosis and NETosis (31, 74). HMGB-1 is cleaved by thrombomodulin–thrombin complexes *in vivo*, and recombinant thrombomodulin is presently approved for the treatment of DIC in Japan and is in phase III trials here in the United States (5, 70).

**Table 1:** **Targets for translation in the prevention of NET-mediated thrombosis (4, 25, 55, 62–70)**.

Scenario	Target	Treatment
Sepsis thrombotic microangiopathies	Histones	Antihistone Antibodies Activated protein C
	cfDNA	DNAse

Endothelial activation and thrombosis (stasis, endothelial injury, coagulopathy, sepsis, trauma, and transplant rejection)	Weibel–Palade body release	Inducers of inducible nitrous oxide synthase (iNOS)
	Platelet α-granule release	Aspirin and clopidogrel thromboxane A2 inhibitors
	vWF	rADAMTS13
	vWF Al domain	vWF Al domain aptamer
	Glycoprotein Ib	Inhibitors of glycoprotein Ib–vWF interaction
	Integrin β2	Inhibitors of integrin β2–vWF interaction
	P-selectin	P-selectin inhibitors and clopidogrel
	HMGB-1	Thrombomodulin
	PAD4	PAD4 inhibitors

Other targets include the components of PMN and platelet recruitment to the endothelium. Specifically, blockade of platelet alpha-granule release or endothelial Weibel–Palade body release would decrease P-selectin and vWF-mediated platelet and PMN recruitment to the endothelium decreasing NETosis (24, 32, 75, 76). Similarly, blockade of P-selectin (24), vWF A1 domain (16), glycoprotein Ib, or integrin beta-2 may reach a similar end, however, more specifically (32). Lastly, the vWF degradation enzyme, ADAMTS13 (a disintegrin and metalloproteinase with thrombospondin type 1 motif – member 13), may be given in a recombinant form (rADAMTS13) to degrade vWF and prevent PMN recruitment with subsequent NETosis (77). Although, each of the above-listed countermeasures may result in mild immunodeficiency, if given in a dose-dependent manner, they may abrogate the pathologic immune-mediated thrombosis without sacrificing immune competence.

Despite the relatively recent discovery of NETs as a contributor to VTE, some long-standing traditional VTE therapies already affect NETs. Polyanionic heparin, long considered the gold standard therapy for prevention and treatment of VTE, has a secondary effect of displacing histones from chromatin (78, 79). This allows increased accessibility of nucleases to the exposed chromatin, further permitting degradation of NETs (78, 79). Similarly, aspirin, recently shown to decrease risk of recurrent VTE (80), inhibits NETosis *in vitro* by inhibition of thromboxane A2 synthesis (63, 81). Finally, clopidogrel has also been shown to decrease inflammation and platelet-mediated expression of soluble P-selectin, further decreasing PMN–platelet interactions and hence NETosis (68). It is currently unknown as to the effects of the new oral anticoagulants on NETs, although this may represent an important area for future investigation.

Thrombolysis has become a key weapon in the arsenal against pathologic thrombosis; however, not all thrombotic events are susceptible to thrombolysis with tissue plasminogen activator (tPA) (82). Indeed, the addition of DNA and histones to a fibrin matrix has been shown to make artificial thrombus more stable, more rigid, and more resistant to tPA, and this is partially remedied by DNase (83). Preliminary data from murine models of VT demonstrate inhibition of thrombus formation with DNase instillation prior to and during IVC stenosis (16, 17). In another study of limb ischemia-reperfusion injury, DNase instillation decreased tissue NETs but did not effect end tissue damage or inflammatory infiltrate (84). In spite of this, more recent studies have shown decreased inflammation, increased tissue perfusion, and improved survival with (1) late DNase treatment in cecal ligation puncture (CLP) model of murine sepsis (55), (2) concurrent DNase and tPA treatment in a rat model of myocardial ischemia (85), and (3) preemptive DNase treatment in a rat model of renal ischemia-reperfusion injury (86). Combination tPA and DNase therapy for thrombolysis of acute ischemic events has yet to be studied in human patients but will likely constitute a major area of research in the near future.

There are still many unanswered questions in the field of NETosis. What are the exact proteomic mechanisms that lead to neutrophil activation and subsequent NET formation? What is the role or RAGE, and does this have implications for diabetics? What is the intracellular cascade that leads to PAD4 induction? After NETosis and thrombosis, how are NETs naturally removed from the resolving thrombus? Do NETs play a role in the post-thrombotic syndrome? Will the addition of DNase to tPA broaden the spectrum of patients that can be treated with thrombolysis as opposed to surgery? These questions and more will be answered with time and continued diligent, meticulous, and conscientious research. We look forward to the future with great expectations for upcoming discoveries in the field of NETosis.

## Author Contributions

Dr. Andrew S. Kimball did the literature review, wrote the paper, and approved of the submission. Drs. Andrea T. Obi and Jose A. Diaz did a literature review, wrote sections of the paper, and approved of the submission. Dr. Peter K. Henke wrote and edited the paper, approved of the submission, and is responsible for the content.

## Conflict of Interest Statement

The authors declare that the research was conducted in the absence of any commercial or financial relationships that could be construed as a potential conflict of interest.
